# In-Air Micro-PIXE Mapping with Chemical Contrast

**DOI:** 10.1021/acs.analchem.5c00943

**Published:** 2025-05-29

**Authors:** Matjaž Kavčič, Ava Rajh

**Affiliations:** † 61790 Jožef Stefan Institute, Jamova 39, Ljubljana 1000, Slovenia; ‡ Faculty of Mathematics and Physics, University of Ljubljana, Jadranska 19, Ljubljana 1000, Slovenia

## Abstract

Currently, chemically specific X-ray fluorescence imaging is restricted to synchrotron facilities and based on the chemical selectivity of X-ray absorption spectra. In this article, we demonstrate the capability of high-energy-resolution micro-PIXE spectroscopy to perform two-dimensional (2D) chemical state mapping of sulfur and phosphorus within spatially inhomogeneous model samples. The approach is based on the parallel-beam wavelength-dispersive tender X-ray emission spectrometer combining polycapillary X-ray optics with diffraction on a flat crystal analyzer, which is used to record chemically sensitive Kβ X-ray emission. This was used to maximize the fluorescence signal of one chemical species vs the other and record 2D maps with pronounced chemical contrast. The ratio of intensities recorded at two preselected X-ray emission energies was used as a unique spectral signature of the particular chemical state to yield pure chemical state maps with high lateral resolution provided by the focused proton beam. The presented approach is not restricted to micro-PIXE but is also applicable to other micro X-ray fluorescence imaging techniques commonly applied in various research fields.

## Graphical abstract

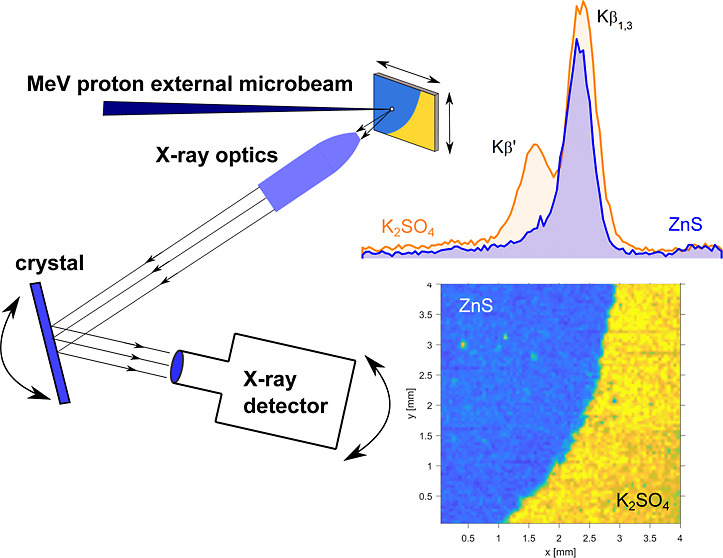

## Introduction

X-ray absorption spectroscopy (XAS) with synchrotron radiation is the most conventional and mature X-ray technique used to perform chemical speciation and study local electronic structure and bonding configuration of atoms within different bulk materials.^1,2^ Combined with a focused incident beam, the near-edge part of the absorption spectrum (XANES) can be applied in microanalysis to record the full distribution of the element under its different oxidation states by recording fluorescence maps at a few preselected excitation energies.^3–6^ However, XAS spectroscopy relies on the use of a monochromatic tunable X-ray beam provided by the synchrotron beamline, so such chemical state mapping is restricted to large-scale synchrotron facilities. Very recently, a Johann-type spectrometer coupled to an X-ray tube has been used to demonstrate the feasibility of such chemical state contrast surface imaging and even tomography also on a laboratory scale.^7,8^ Currently, this is limited to transmission mode measurements, and the lateral resolution is defined by the pixel size of the 2D detector.

Complementary to XAS, the local electronic structure of bulk materials can be studied with high-energy resolution X-ray emission spectroscopy (XES). Generally, emission spectra are independent of the excitation mode, and different laboratory sources of ionizing radiation can be effectively used to produce the initial core-hole state, making XES well suited for laboratory in-house experiments. Particularly in the tender X-ray range covering K absorption edges of light elements (Al, P, S, Cl), XES might be the most viable option for such laboratory analysis since modern laboratory XAS setups typically operate in transmission mode and their use is restricted to the hard X-ray range.^7,9–13^ Several laboratory tender XES spectrometers have been introduced recently^14–16^ showing very good analytical capabilities. The spectrometers provide high energy resolution on the level of core-hole lifetime broadening of the measured emission lines and measured XES spectra exhibit a high degree of chemical sensitivity, which is used to perform electronic structure studies of different bulk materials.^17–20^ Common to these laboratory tender XES spectrometers is a dispersive mode of operation employing a position-sensitive X-ray detector to simultaneously record the full emission spectrum. This is not applicable for chemical state mapping, as it is not feasible to record a series of full XES spectra from a large raster of microspots across a sample surface requiring short collection times in the order of sec/point. For that purpose, a scanning-type spectrometer providing much higher solid angle collection efficiency would be preferable and might be used to perform chemical imaging by collecting fluorescence at certain preselected emission energies in direct analogy with the XAS imaging approach used at synchrotron facilities.

In our laboratory, X-ray emission induced with a microfocused MeV proton beam (micro-PIXE) is used to quantitatively analyze spatial distribution of minor and trace elements in the samples with a micrometer lateral resolution.^21–24^ Complementary to energy dispersive detectors (EDS) used in conventional PIXE analysis, a new parallel beam wavelength dispersive (PB-WDS) X-ray emission spectrometer has been recently installed at our external proton microbeam to improve the energy resolution of fluorescence detection.^25^ The divergent X-rays emitted from a point source defined by the microfocused incident beam on the sample are collected over a large solid angle by the polycapillary X-ray optics and converted into a collimated X-ray beam, which is diffracted by a flat crystal analyzer. The energy resolution of the spectrometer is primarily defined by the divergence of the polycapillary lens and reaches a few eV, which is an order of magnitude better than the resolution obtained with the EDS detectors. This is primarily used to completely remove or reduce the overlaps between particular X-ray lines in the measured PIXE spectra, which in many cases hinders the analysis of a particular element in the sample.

Recently, it was shown that the spectrometer is capable of performing also the chemical state analysis of certain low-Z elements.^26^ A significant chemical contrast was achieved in the measured Kβ X-ray emission spectra of P and S, reflecting the structure of occupied valence molecular orbitals defined by the first coordination shell around the central atom. In this work, we further exploit this feature by mapping a distribution of different P and S chemical species within a spatially inhomogeneous sample. Two different model samples were prepared containing S and P in different chemical states, and the spatial distribution of each chemical species has been recorded, demonstrating the feasibility of such chemical state micro-PIXE mapping.

## Experiment

The experiment was performed at the external beamline of the 2MV Tandem accelerator at the Microanalytical Center of the J. Stefan Institute (JSI). Protons were accelerated to 3 MeV and focused with a doublet of magnetic quadrupole lenses. The focused proton beam was extracted into air through a beamline exit nozzle closed with a 200 nm Si_3_N_4_ window and directed onto a sample mounted on the motorized computer-controlled XYZ target stage, which was used to position the sample and move it across the beam. The sample was positioned close to the exit nozzle at a distance of a few millimeters to minimize the lateral straggling of protons in their passage through the air gap between the exit window and the sample and achieve good lateral resolution. The final beamspot size on the sample surface was ∼50 × 50 μm^2^, and the incident proton current was ∼20 nA. MeV proton irradiation at high current density might induce chemical state changes, and this has been observed and systematically studied, particularly for solid sulfur samples.^27,28^ The sulfate/phosphate compounds with closed shell structure used in our work are expected to be chemically stable against proton irradiation, and the same holds also for the sulfide/phosphide compounds. This was checked during the accumulation of the full Kβ emission spectra of P and S from different fixed sample spots. In this case, several consecutive scans were performed, and no changes between spectra accumulated in separate single scans have been observed. While no significant radiation damage has been observed in our particular study, it might represent an important issue in the general applicability of this approach combined with MeV focused ion beams.

Proton-induced X-ray emission spectra were recorded with the PB-WDS spectrometer installed recently at the beamline.^25^ The spectrometer starts with the polycapillary X-ray semilens positioned at 45 ° relative to the incident beam and 10 mm from the sample surface, corresponding to the focal length of the lens. A collimated X-ray beam exiting the polycapillary optics was diffracted by a Ge(111) flat crystal analyzer and finally collected with a 25 mm^2^ Si-PIN diode. The full Kβ emission spectra of P and S were recorded in a point-by-point scanning mode. Energy calibration of the spectrometer was performed using the Kβ_1,3_ reference emission energies of phosphate^29^ and sulfate^30^ material. During the PIXE mapping, the Bragg angle of the spectrometer was adjusted to one fixed value corresponding to a particular preselected emission energy, and the X-ray yield was recorded as a function of the sample position, which was raster scanned across the focused proton beam by the motorized sample stage. Simultaneously with the X-ray emission, the spectrum of the backscattered protons from the rotating chopper (gold-plated carbon blades) periodically intersecting the beam was recorded, and the integrated Au Rutherford backscattering spectrometry (RBS) peak was used to perform normalization for the accumulated proton dose. A schematic view and a photo of the experimental setup with the PB-WDS spectrometer are shown in Figure 1.

**Figure 1:**
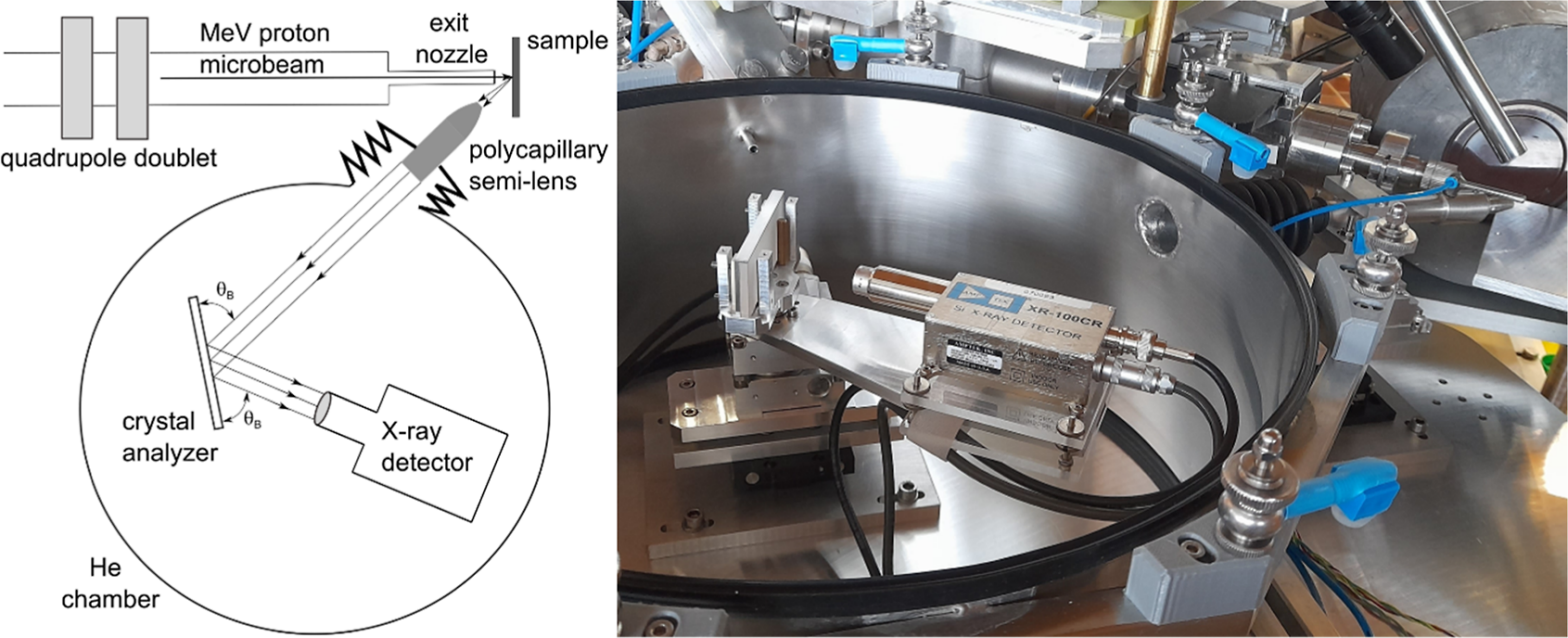
(left) Scheme (not to scale) and photo (right) of the PB-WDS setup installed at the external proton beamline.

## Results and Discussion

A simple model target was prepared with two spatially well-separated regions of different sulfur species. Sulfate (K_2_SO_4_) and sulfide (ZnS) powders were used. First, a pellet from ZnS powder was pressed, and then it was encased in a layer of K_2_SO_4_ and pressed again to merge the two materials into a final sample with a well-defined boundary between both regions. Figure 2. The actual distribution of each sulfur species within the model sample obtained from the standard PIXE maps of K and Zn recorded with the Si­(Li) detector is shown in Figure 3a.

**Figure 2:**
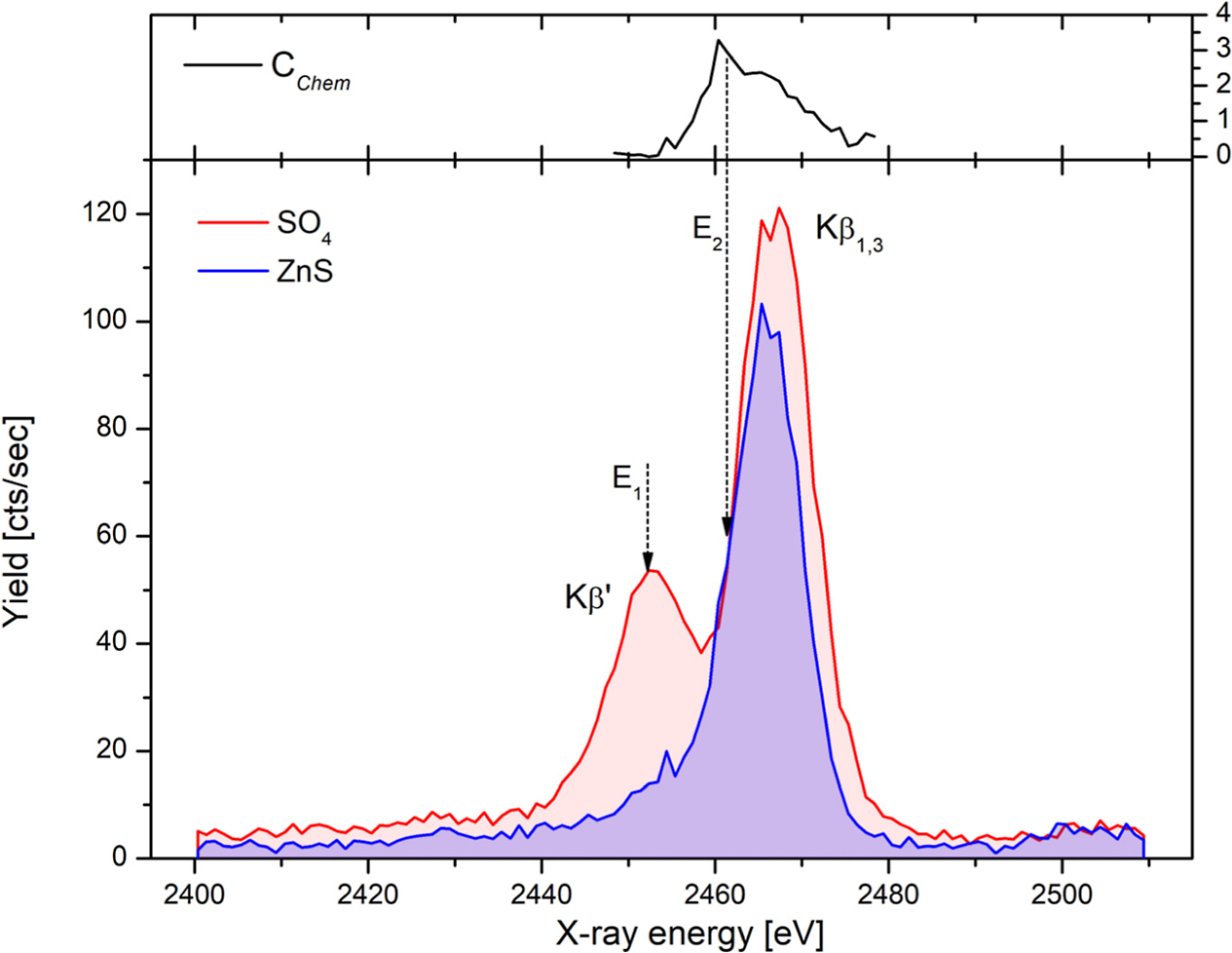
Sulfur Kβ emission spectra recorded by the PB-WDS spectrometer from separate regions of the model sample. The same incident proton dose was delivered to the sample for both measured spectra. The upper part of the figure shows the chemical contrast as a function of X-ray energy used to select the second emission energy in PIXE mapping, as discussed later on in the text.

**Figure 3:**
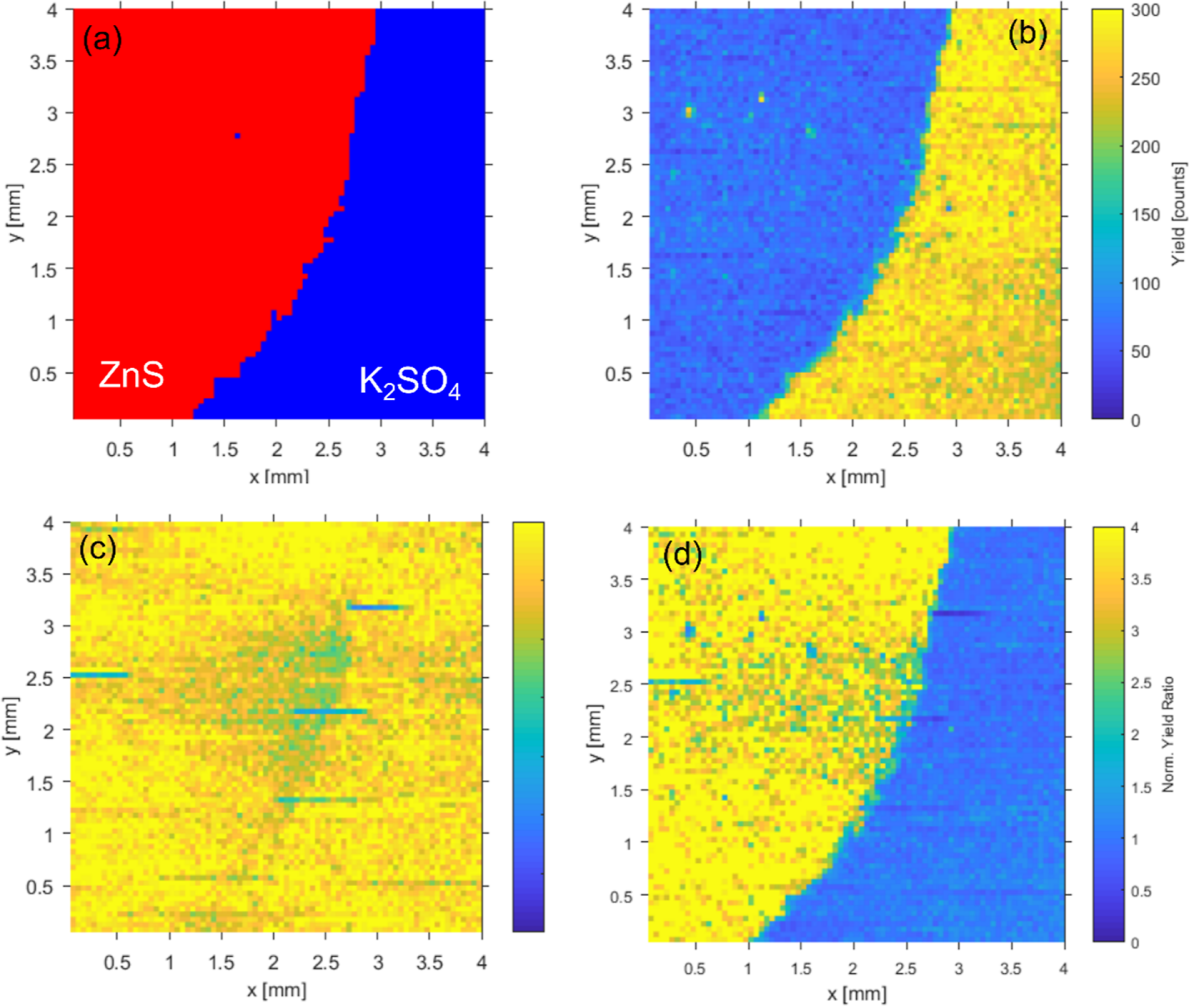
(a) Actual ZnS/K_2_SO_4_ distribution within the model sample obtained from the standard Zn/K PIXE maps recorded with the Si­(Li) detector. (b) Sulfur micro-PIXE map recorded with the PB-WDS spectrometer tuned to the energy of the Kβ′ component in the SO_4_ spectrum. The raster scan consisted of 80 × 80 points, with a step size of 50 μm and an acquisition time of 5 s per point. (c) Sulfur micro-PIXE map recorded with the PB-WDS spectrometer tuned to the second emission energy E_2_. (d) Yield ratio R­(E_2_,E_Kβ’_) obtained from micro-PIXE maps recorded with PB-WDS at both emission energies. For clarity, yield ratio R is normalized to the average value recorded within the sample region corresponding to K_2_SO_4_.

First, the S Kβ emission spectra from each part of the sample were recorded by scanning the PB-WDS spectrometer over the 2400–2510 eV energy interval with a 1 eV step. The acquisition time for a single point was 5 s; 6 separate consecutive scans were performed and summed up to reach the final spectrum. Both measured spectra are shown in Figure 2. Despite the moderate energy resolution of the PB-WDS spectrometer (6.6 eV ± 0.6 eV at S Kα line^25^), good chemical sensitivity is observed in the measured spectra. While the SO_4_ spectrum exhibit two pronounced spectral components Kβ_1,3_ and Kβ′ reflecting the structure of occupied valence molecular orbitals defined by the tetrahedral molecular geometry of the SO_4_^2–^ ion,^31^ the Kβ′ component is absent from the spectrum of ZnS with direct bonding of metal ligand to sulfur atom.

Next, the spectrometer has been set to the Bragg angle corresponding to the energy of the Kβ′ component in the SO_4_ spectrum (E_1_ at Figure 2), where the ratio of the recorded signal between the two S species is maximal, and the X-ray intensity was recorded as a function of a sample position, which was raster scanned across the focused proton beam. The recorded intensity map is presented in Figure 3b, clearly separating regions with different sulfur species. The chemical contrast is in this case additionally enhanced due to differences in the sulfur content, proton stopping power, and target self-absorption, all together yielding different overall sulfur X-ray fluorescence from both sample regions. This is observed directly in Figure 2, where the total integrated Kβ emission yield of K_2_SO_4_ is larger compared to the one of ZnS. However, the contrast in the measured map is significantly higher than the measured overall K_2_SO_4_/ZnS Kβ yield ratio.

While the S map recorded with the PB-WDS tuned to E_1_ exhibits the SO_4_ distribution in the sample with good contrast, it is not possible to find a single emission energy to enhance the ZnS signal over the SO_4_ one and record in the same way also the ZnS distribution. To get a pure chemical state signal independent of the overall sulfur fluorescence, a full Kβ emission spectrum should be recorded at each point. As already explained in the introduction, this is experimentally practically not feasible due to the short acquisition times available for each single spot on the sample surface. The time restriction is reduced drastically by using the relative intensity ratio approach proposed recently.^32^ Here the ratio of intensities *R*_i_(*E*_1_ *,E*_2_) = *Y*_i_(*E*_1_)/*Y*_i_(*E*_2_) recorded at two different appropriately selected emission energies within the spectrum is used as a pure spectral signature of the chemical state of an element within the sample. In our particular case, the choice of the first emission energy at the top of the Kβ′ component is obvious. The intensity ratio as a function of second emission energy *E*_*X*_

1 $$R_{i}\left(E_{X},E_{K\beta^{\prime }}\right)=\frac{Y_{i}\left(E_{X}\right)}{Y_{i}\left(E_{K\beta^{\prime }}\right)}$$

is a unique quantity depending only on the spectral shape defined by the sulfur chemical state and therefore free from any other experimental effects discussed previously. The chemical contrast between both species *i, j* is now defined as a relative difference of the corresponding intensity ratios

2 $$C_{Chem}\left(E_{X}\right)=\frac{\left|R_{i}\left(E_{X},E_{K\beta^{\prime }}\right)-R_{j}\left(E_{X},E_{K\beta^{\prime }}\right)\right|}{\min\left(R_{i}\left(E_{X},E_{K\beta^{\prime }}\right),R_{j}\left(E_{X},E_{K\beta^{\prime }}\right)\right)}$$

Using both measured Kβ emission spectra, this contrast can be plotted as a function of second emission energy *E*_*X*_, as shown in the top part of Figure 2, exhibiting a maximum somewhere around the middle of the low-energy slope of the main Kβ_1,3_ component. Based on this, another map (shown in Figure 3c) has been recorded with the spectrometer set to the second emission energy E_2_ indicated in Figure 2, where the highest relative difference of the corresponding intensity ratios (chemical contrast *C*_Chem_ defined by eq 2) between both species is expected. Despite the almost homogeneous distribution of the S signal recorded at E_2_, the intensity ratio obtained from both measured maps normalized to the same incident proton dose can be used to separate contributions from each of the two species and finally obtain their spatial distributions. For example, the *R*(*E*_*2*_,*E*_Kβ’_) map is plotted in Figure 3d, exhibiting the distribution of ZnS within the model sample despite its lower overall fluorescence compared to the SO_4_ one. This is now a pure chemical state map; the contrast of such chemical state PIXE mapping is provided by the energy resolution of the spectrometer solely, and the lateral spatial resolution is given by the size of the focused proton beam.

In the second case, we have applied the same principle also for chemical state mapping of phosphorus within a structurally slightly more complex sample. For that purpose, a model sample was prepared containing an inhomogeneous mixture of two different phosphorus species, namely Fe_2_P and NaH_2_PO_4_. The photo of the sample seen under the microscope is presented in Figure 4.

**Figure 4:**
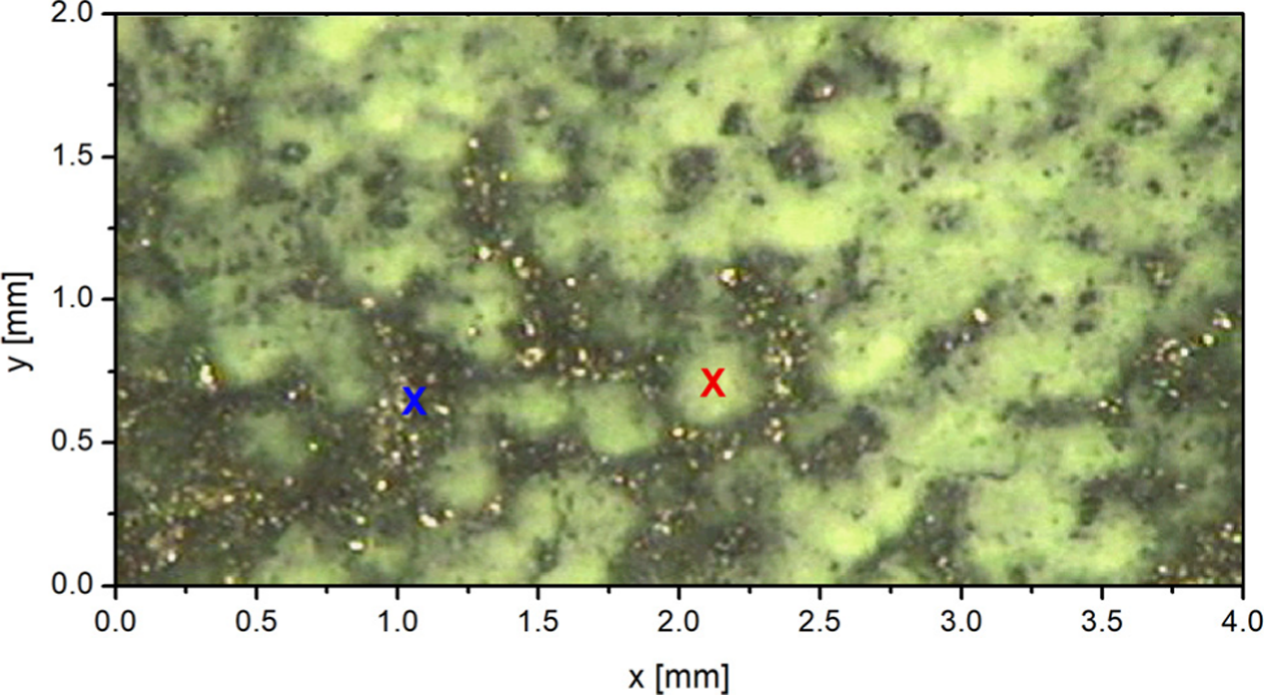
Photo of the Fe_2_P/PO_4_ model sample used to perform phosphorus chemical state mapping. The sample was prepared as a pellet pressed from inhomogeneous mixture of pure Fe_2_P and NaH_2_PO_4_ powders. Due to color difference, the white regions of predominantly phosphate species are distinct from the darker ones containing mainly Fe_2_P. The crosses indicate the spots used to record the full Kβ emission spectra.

The Kβ emission spectra were recorded with a PB-WDS spectrometer from two different spots on the sample selected within the white and dark regions, respectively. The energy interval 2093–2172 eV around the P Kβ_1,3_ diagram line has been scanned with the spectrometer in steps of 0.5 eV and 5 s acquisition time per single point. Three separate consecutive scans were performed and summed up; both final recorded spectra are presented in Figure 5. Going from sulfur to phosphorus emission energies, the energy resolution of the spectrometer is increased (3.5 eV ± 0.3 eV at P Kα line^25^), and the measured spectra exhibit high contrast between both chemical species. Next, the mapping has been performed by tuning the spectrometer to the energy of the Kβ′ component and recording the X-ray intensity as a function of a sample position, which was raster scanned across the proton beam. The recorded map is shown in Figure 6a.

**Figure 5:**
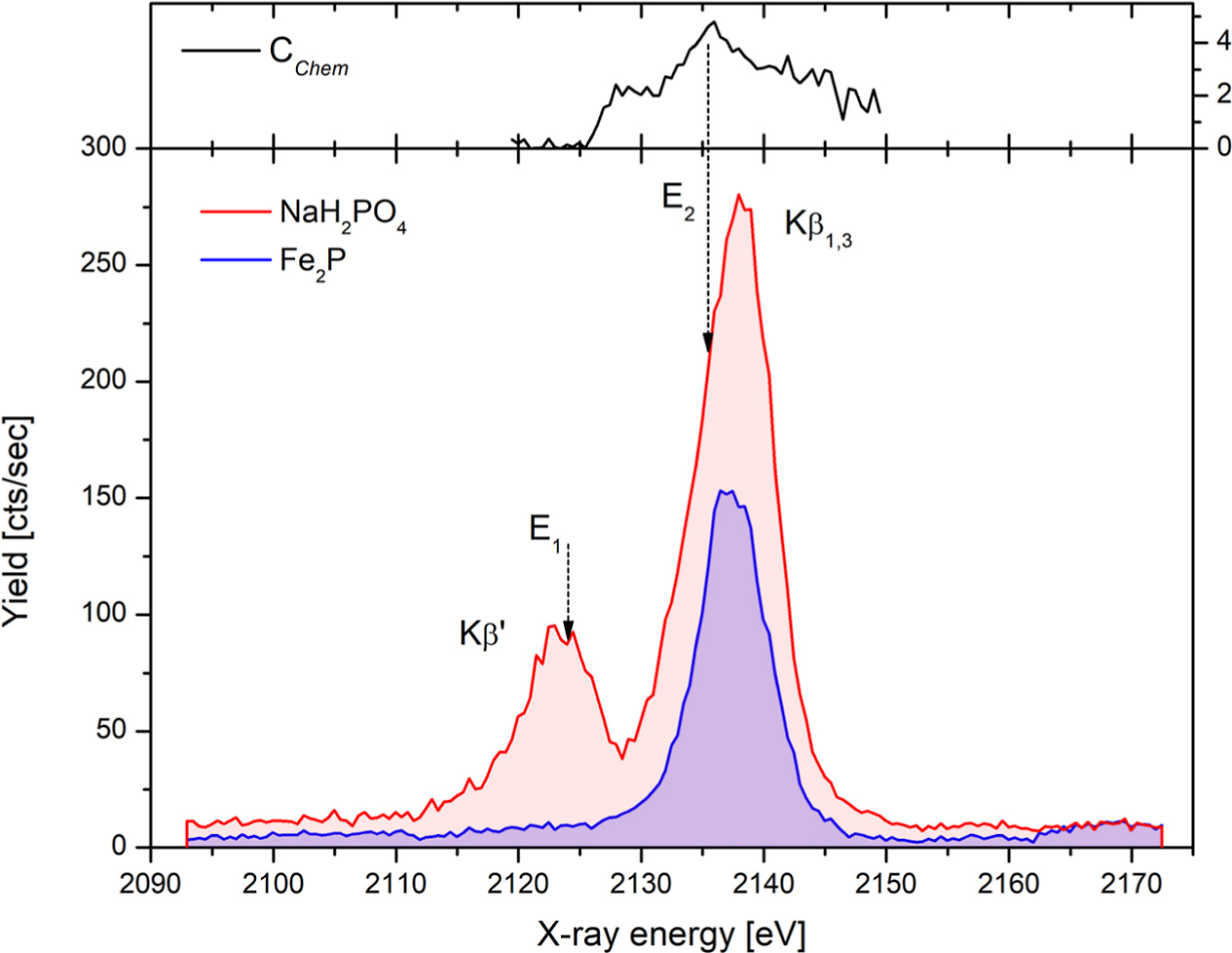
Phosphorus Kβ emission spectra recorded by the PB-WDS spectrometer from two selected spots on the model sample. Spectra were normalized to the same incident proton dose. Both emission energies used later in PIXE mapping are also indicated.

**Figure 6:**
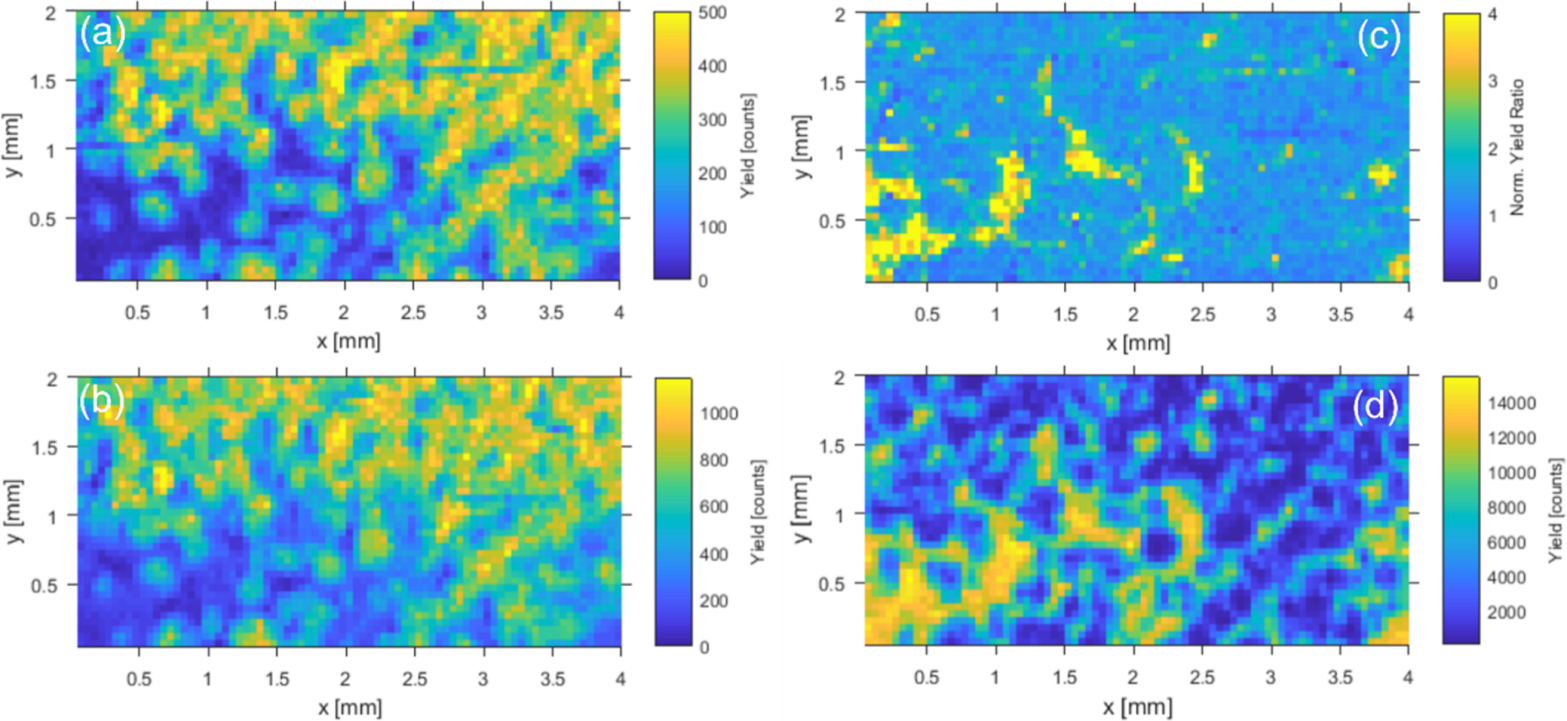
(a) Phosphorus micro-PIXE map recorded with the PB-WDS spectrometer tuned to the energy of the Kβ′ component in the PO_4_ spectrum. The raster scan consisted of 80 × 40 points, with a step size of 50 μm and an acquisition time of 5 s per point. (b) Phosphorus micro-PIXE map recorded with the PB-WDS spectrometer tuned to the energy *E*_2_. (c) Normalized yield ratio *R*(*E*_2_,*E*_Kβ’_) obtained from phosphorus micro-PIXE maps measured at both emission energies. (d) Standard Fe PIXE map recorded with the Si­(Li) detector exhibiting the Fe_2_P regions.

The PO_4_ X-ray emission intensity recorded with the PB-WDS spectrometer set to *E*_Kβ’_ energy is practically an order of magnitude higher than the corresponding Fe_2_P intensity. Consequently, the recorded map exhibits, with high contrast, the distribution of PO_4_ species within the model sample. The spatial resolution is defined by the focused proton microbeam, and a nice correspondence with the optical image of the sample (Figure 4) is observed. As already explained previously, the very high PO_4_/Fe_2_P contrast observed in Figure 6a is not entirely due to the chemical sensitivity but is additionally enhanced by the difference in the overall phosphorus K X-ray fluorescence of the two chemical species, which is seen in the measured Kβ emission spectra on Figure 5. To get the pure chemical state map, the same procedure used before for S has been followed also for the P sample. The spectrometer was tuned to the second emission energy (*E*_2_ shown in Figure 5), which was selected to reach the highest relative difference of the corresponding intensity ratios (*C*_Chem_ defined by eq 2) between both P species, and another map has been recorded, shown in Figure 6b. The ratio of both recorded signals is now used as a pure chemical state spectral signature, and the final map is plotted in Figure 6c. For comparison, the standard Fe PIXE map recorded with the Si­(Li) detector is shown in Figure 6d, corresponding to the actual distribution of Fe_2_P in the model sample. Compared to that in Figure 6a, the contrast in the recorded intensity ratio map is reduced, as expected. However, we are left with the pure P chemical state map. The major large area uniform spots of each species are clearly observed in the map, while some minor fine structure details, which are resolved in Figure 6a,d, are smeared out. This is attributed to the normalization of the proton dose accumulated at each separate point on the sample surface during the two consecutive maps. Because of the relatively low count rate of protons backscattered from the chopper, which is used for normalization for the proton dose, we needed to sum the integrated Au RBS signal recorded during a few tens of neighboring points on the sample to reach low enough statistical uncertainty of accumulated Au RBS yield. While this was good enough to correct for the slow continuous variations of the incident proton current, possible smaller discrete fluctuations between separate points were not accounted for. Within the next step, we are going to adjust the mechanical assembly of our chopper to increase the solid angle of the RBS detector and increase the count rate, which will allow for full proper normalization of each point of the recorded map. In principle, this problem could be avoided by using two spectrometers in parallel, recording X-ray intensity at both preselected emission energies simultaneously. In such a case, the pure chemical state map could be recorded within a single raster scan, and any problems related to the proton dose normalization would be removed completely.

## Conclusions

In this study, we exploited a combination of an MeV external proton microbeam and a parallel-beam wavelength-dispersive X-ray spectrometer to record 2D chemical state maps of sulfur and phosphorus within spatially inhomogeneous model samples. The experimental concept presented here is based on the new high-energy-resolution spectrometer providing chemical sensitivity and high enough collection efficiency to be used for 2D mapping. The proposed method is particularly suitable for third period elements (P, S, Cl), using intense Kβ diagram fluorescence lines exhibiting high chemical sensitivity.^31,33,34^ Using this approach, the chemical state mapping is not restricted to large-scale synchrotron facilities and X-ray absorption spectroscopy but can be successfully transferred to smaller laboratories equipped with microfocused ionization probes. The results presented here serve as a proof of concept by using basic model samples composed of species with pronounced chemical contrast. Generally, this contrast varies from one compound to another; it also depends on the energy resolution of the spectrometer and should therefore be studied on a case-by-case basis. However, these results confirm the general feasibility of the micro-PIXE 2D chemical state mapping with possible applications in the fields of biology, energy storage materials, and archeometry, all regularly exploiting micro X-ray fluorescence 2D mapping techniques.
